# Hepatitis A outbreak among men who have sex with men (MSM) predominantly linked with the EuroPride, the Netherlands, July 2016 to February 2017

**DOI:** 10.2807/1560-7917.ES.2017.22.8.30468

**Published:** 2017-02-23

**Authors:** Gudrun S Freidl, Gerard JB Sonder, Lian PMJ Bovée, Ingrid HM Friesema, Gini GC van Rijckevorsel, Wilhelmina LM Ruijs, Frank van Schie, Evelien C Siedenburg, Jyh-Yuan Yang, Harry Vennema

**Affiliations:** 1 Centre for Infectious Diseases, Epidemiology and Surveillance, Centre for Infectious Disease Control, National Institute for Public Health and the Environment (RIVM), Bilthoven, the Netherlands; 2 European Programme for Intervention Epidemiology Training (EPIET), European Centre for Disease Prevention and Control (ECDC), Stockholm, Sweden; 3 Department of Infectious Disease Control, Public Health Service Amsterdam (GGD), Amsterdam, the Netherlands; 4 National Coordination Centre for Communicable Disease Control, Centre for Infectious Disease Control, National Institute for Public Health and the Environment (RIVM), Bilthoven, the Netherlands; 5 Centers for Infectious Disease Control, Taipei, Taiwan; 6 Centre for Infectious Diseases Research, Diagnostics and Screening, Centre for Infectious Disease Control, National Institute for Public Health and the Environment (RIVM), Bilthoven, the Netherlands

**Keywords:** hepatitis A, men who have sex with men – MSM, The Netherlands, sexually transmitted infections, viral infections, mass gatherings, outbreaks, epidemiology, surveillance

## Abstract

Between July 2016 and February 2017, 48 male cases of hepatitis A were notified in the Netherlands. Of these, 17 identified as men who have sex with men (MSM). Ten of the 13 cases for whom sequencing information was available, were infected with a strain linked with the EuroPride that took place in Amsterdam in 2016. This strain is identical to a strain that has been causing a large outbreak among MSM in Taiwan.

In December 2016, the European Centre for Disease Prevention and Control (ECDC) issued a Rapid Risk Assessment reporting of two distinct hepatitis A virus (HAV) genotype IA strains circulating among men who have sex with men (MSM) in the United Kingdom (UK) and the Netherlands in 2016. Germany, Italy and Spain had also reported a recent increase in male HAV cases [1].

The outbreak is ongoing with 37 cases reported in the UK between July 2016 and January 2017 [2] and 30 cases in Berlin between mid-November 2016 and end of January 2017 [3]. Here we describe the current situation in the Netherlands including potential links to this international hepatitis A outbreak.

## Case definition

A case was defined as a person who (i) met the surveillance definition of a case of hepatitis A, (ii) self-identified as MSM or had MSM contact i.e. sexual contact with another man two months before the onset of symptoms, (iii) developed symptoms after mid-2016 (3 July 2016) and (iv) was a resident in the Netherlands. The surveillance case definition comprises (i) non-specific symptoms (e.g. fatigue, abdominal pain, loss of appetite, intermittent nausea and vomiting), (ii) fever or jaundice and (iii) laboratory confirmation or an epidemiological link with a laboratory-confirmed case i.e. either hepatitis A-specific IgM antibodies in serum or detection of HAV in serum or stool by means of PCR [4].

## Surveillance of hepatitis A in the Netherlands

In the Netherlands, hepatitis A is a notifiable disease. Laboratories and physicians report HAV infections within one working day to the regional Public Health Services (PHS). The PHS then collect epidemiological information on demographics, occupation, symptoms, suspected source / country of infection, MSM contact (for males only) and consumption of specific food items. The PHS reports all information in the national surveillance system for notifiable diseases. In addition, serum and / or stool samples of HAV cases are routinely sent to the National Institute of Public Health and the Environment (RIVM) for molecular analysis. In case men did not explicitly report having had MSM contact two months before disease onset, MSM status was assessed by asking whether they identified themselves as MSM.

## Molecular analyses

HAV IgM-positive serum samples were analysed by sequence analysis of a 460 nt PCR fragment in the VP1/P2A region according to a shared protocol available through Hepatitis A Lab-Network HAVNET [5].

## Outbreak description

In the first half of 2016 (including week 26), 22 sporadic hepatitis A cases were notified through the Dutch national surveillance system. Half of these were men and none reported MSM contact.

On 19 September 2016 (week 38), the outbreak investigation was triggered by the notification of two male cases of hepatitis A, in their 30s and 40s, who fell ill in mid-September. Both cases reported having had MSM contact during the EuroPride. The EuroPride, which took place in Amsterdam between 29 July and 6 August, is an international event to celebrate equality rights of the lesbian, gay, bisexual and transgender community. In 2016, this event attracted over half a million visitors [6]. Sequencing showed that strains from both cases were identical (RIVM-HAV16–090). Given the international character of the EuroPride, alerts were placed on the Early Warning and Response System (EWRS) and on ECDC’s Epidemic Intelligence Information System for Food- and Waterborne diseases (EPIS-FWD) to inform other European countries.

From mid-2016 (week 27) to 7 February 2017, 48 male cases of hepatitis A were reported nationally. Of these, 17 identified as MSM. Two cases did not (yet) meet the case definition, as MSM status was unknown at the time of the investigation. For comparison, in 2013, 2014 and 2015, 56, 58 and 45 male cases of hepatitis A were reported each year, respectively. Among these, none were identified as MSM.

**Figure 1:**
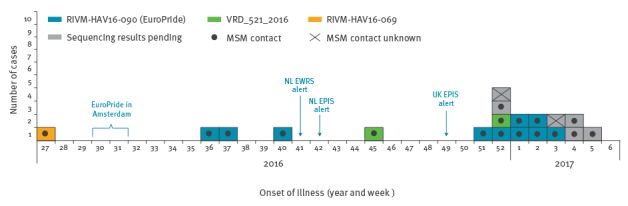
Epidemic curve of hepatitis A cases by MSM status and week of onset of illness, July 2016–February 2017, the Netherlands (n = 19)

EPIS: Epidemic Intelligence Information System; EWRS: Early Warning and Response System; MSM: men who have sex with men; NL: the Netherlands; UK: United Kingdom.

The Table shows the characteristics of the cases recorded in the current outbreak. The onset of illness ranged from week 27, 2016 to week 5, 2017 (Figure 1).

**Table:** Characteristics of hepatitis A cases by MSM status and strain, the Netherlands, July 2016–February 2017 (n = 19)

	Men who have sex with men (MSM)					Sequence information and MSM status unknown / pending
**Hepatitis A strain**	RIVM‑HAV16‑090 (EuroPride)	VRD_521_2016 (UK / Spain)	RIVM-HAV16–069	Sequence information pending	Total	
**Number of cases**	10	2	1	4	17	2
**Number per 10-year age group**	20–29 (n = 3) 30–39 (n = 4) 40–49 (n = 3)	20–29 (n = 1) 40–49 (n = 1)	20–29 (n = 1)	20–29 (n = 2) 30–39 (n = 1) 50–59 (n = 1)	33 (26–52) Median (min–max)	20–29 (n = 1) 40–49 (n = 1)
**Number of cases hospitalised**	3	1	1	0	5	0
**Number of cases vaccinated against hepatitis A**	0	0	0	0	0	0
**Suspected place of infection**	The Netherlands (n = 8) Spain, Barcelona (n = 2)	The Netherlands (n = 1) Spain (n = 1)	Argentina (n = 1)	The Netherlands (n = 3)^a^ Germany, Berlin (n = 1)	4	The Netherlands (n = 1)^b^

MSM: men who have sex with men; UK: United Kingdom.

^a^ One case reported Portugal as second suspected country of infection.

^b^ For one case information on suspected place of infection was not available.

Ages were estimated because only year of birth was known.

Characteristics of two male cases of hepatitis A for whom MSM status was unknown at the time of the investigation, are shown separately.

Two cases for whom MSM status was unknown at the time of the investigation, are also included.

**Figure 2:**
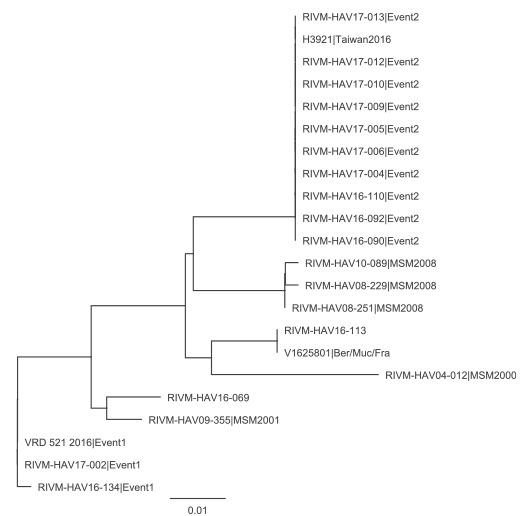
Phylogenetic analysis of virus strains from hepatitis A cases who self-identified as men who have sex with men, the Netherlands, 2000–2017

Of the 17 cases, 11 were born outside the Netherlands (Argentina, Brazil, Canada, France, Italy, Lebanon, Peru, Spain (n = 3), Surinam). The median age of the 17 cases was 33 years (range: 26–52). None of the cases were vaccinated and about a third was hospitalised (Table). Sequence information was available for 13 of the 17 cases, which showed co-circulation of three different hepatitis A strains (Table, Figure 2).

Neighbour-joining tree of sequences of 445 nt of the VP1/P2A region of hepatitis A virus strains.

The tree was constructed in PHYLIP (DNADIST) (Joe Felsenstein, Department of Genome Sciences, University of Washington, Seattle, USA). Strains are identified by sample number and cluster. Event 1 and Event 2 refer to terminology used in the Rapid Risk Assessment published by the European Centre for Disease Prevention and Control (ECDC) [1]. Event 1 refers to strains detected in the United Kingdom and Spain [2]. Event 2 refers to the EuroPride strain detected in the Netherlands and the strain circulating in Taiwan in 2016, respectively. One asymptomatic case is identical to a sequence that caused a cluster in Germany (Ber/Muc/Fra) [3]. For comparison, we also included older hepatitis A virus strains detected in men who have sex with men (MSM) in 2000, 2001 and 2008, respectively.

**Figure 3:**
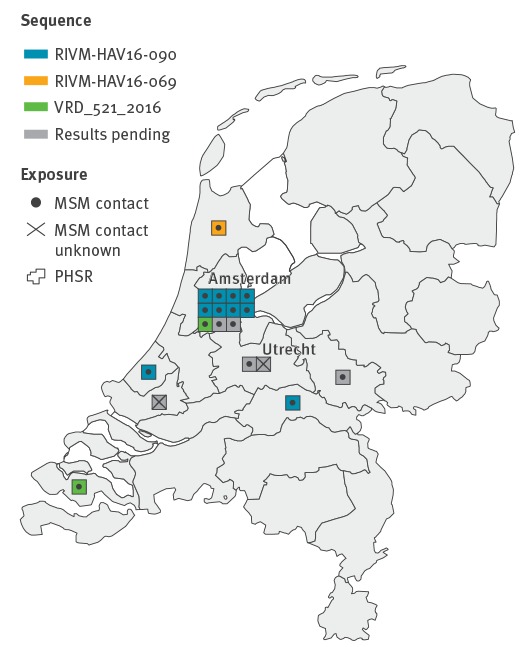
Geographic distribution of hepatitis A cases who self-identified as men who have sex with men, by available sequence information, the Netherlands, July 2016–February 2017 (n = 19)

MSM: men who have sex with men; PHSR: Public Health Service region.

Ten of the 13 cases with available typing information were infected with the EuroPride strain. The majority of cases (n = 11), irrespective of sequence type, clustered in the Public Health Service region of Amsterdam, whereas other Public Health Service regions only reported incidental cases (Table, Figure 3).

Two cases for whom MSM status was unknown at the time of the investigation, are also shown. Notified cases are centred in the respective Public Health Service region. The majority of cases (n = 11) occurred in the PHSR Amsterdam.

In comparison, among the 29 male cases who became ill after mid-2016 and were not MSM (median age: 20.5 years, range: 0–82) we found strains that were unrelated to the current outbreak. We detected genotype IA and IB strains from Morocco, IB strains from Egypt, Turkey, West Africa and East Africa, a IIA strain from Cameroon, a IIIA strain from Romania or no hepatitis A virus, respectively. As none of these cases was infected with a strain involved in the current outbreak, we are confident that these cases reported their MSM status truthfully.

## EuroPride strain RIVM-HAV16–090

When comparing sequence information of the EuroPride strain with available sequences in the databases HAVNET [5] and GenBank, we found that the EuroPride strain was 99.57% identical to a sequence submitted by Japan (accession number: AB020565, release date: 14 August 2001). In addition, in response to a post on ProMED-mail from May 2016 that reported on a hepatitis A outbreak among MSM in Taiwan with 275 notified cases [7], we investigated whether the EuroPride strain might be related to the Taiwanese outbreak strain. Direct comparison and phylogenetic analyses showed that the Taiwanese outbreak strain was identical to the EuroPride strain (Figure 2). Eight of the ten cases reported to have likely been infected in the Netherlands, and a further two cases were likely infected in Barcelona, Spain (n = 2; onset of illness for both cases: week 2, Figure 1).

## Strains VRD_521_2016 and RIVM-HAV16–069

Two cases were infected with strain VRD_521_2016, first reported by the UK in December 2016 and likely imported from Spain several times [1,2]. One of the Dutch cases reported having travelled to Spain (onset of illness in week 45), whereas the other case stated to have likely been infected in the Netherlands (onset of illness week 52, Figure 1).

One case infected with strain RIVM-HAV16–069 reported having travelled to Argentina and became ill shortly before the EuroPride (week 27). The UK also reported one MSM case with the same sequence [2].

## Discussion

Here we report on an ongoing hepatitis A outbreak among MSM in the Netherlands that started in 2016. Hepatitis A is an acute, self-limiting liver disease which is transmitted via the faecal-oral route. Infection occurs via contaminated food or water, or through person-to-person contact, including sexual contact. The average incubation period is 28 days (range: 15–50 days) [8]. In western Europe hepatitis A endemicity is low [9] and is primarily associated with travelling to endemic countries [10] or consumption of contaminated, imported food [11]. Outbreaks among MSM have also been described [12]. In Europe, the last outbreak of hepatitis A among MSM occurred between 2008 and 2011 [13]. Between 2012 and mid-2016, hepatitis A infection in MSM was only notified twice in the Netherlands.

In the currently ongoing outbreak in the Netherlands, the majority of cases for whom sequence information was available, were infected with strain RIVM-HAV16–090. This strain had only been detected once before in 2010 and was absent in the Netherlands until it was detected in two MSM cases who attended the EuroPride in 2016. The strain is identical with a strain causing an ongoing outbreak among MSM in Taiwan. As at 29 September 2016, Taiwan reported 845 hepatitis A cases among MSM, of which 56% were HIV-positive or had another sexually transmitted diseases [14].

In the Netherlands, information on HIV status is not routinely collected for hepatitis A surveillance purposes. In the course of this outbreak investigation, in week 43, we detected one HAV infection in a HIV-positive MSM who was asymptomatic and therefore did not meet the case definition. Sequencing showed infection with a strain identical to the Berlin/Munich/Frankfurt HAV cluster in Germany (V16–25801) [3]. Asymptomatic individuals, even if they do not fulfil the case definition, can still be epidemiologically relevant and should therefore be included in epidemiological analyses.

In the Netherlands, besides risk groups, i.e. persons with chronic liver disease or occupational exposure to HAV, hepatitis A vaccination is recommended to individuals who travel to HAV endemic countries. Hepatitis A vaccine uptake is unknown. Because of several outbreaks among European and Dutch MSM [15,16], hepatitis A vaccination is also recommended to MSM in the Netherlands. For MSM, vaccination against HAV is available at reduced costs and is administered in combination with hepatitis B vaccine that is free of charge for this risk group. The uptake of hepatitis B vaccination among MSM in Amsterdam is high and hepatitis B incidence has dropped markedly since 2005 [17]. In contrast, financial aspects might hamper wide uptake of hepatitis A vaccination. Vaccination coverage among MSM is unknown.

In the Netherlands, hepatitis A control is based on vaccination of household- and other close contacts [4]. Tracing and vaccination of sexual contacts of MSM can be challenging due to anonymous sexual contacts. To better understand transmission chains and the epidemiology of this outbreak, we recently introduced an additional, more detailed questionnaire for hepatitis A-positive MSM to complement routinely collected epidemiological data. Given the high outbreak potential of hepatitis A in the MSM community and the high interconnectedness through global travel of this risk group [18], increasing awareness of hepatitis A among MSM as well as health professionals at sexually transmitted disease clinics and public health services should be emphasised. To increase hepatitis A vaccination uptake, the Regional Public Health Services and ‘STI AIDS the Netherlands’ (centre of expertise for HIV and other sexually transmitted infections) have been engaging in activities to remind professionals and the Dutch MSM community of the availability of hepatitis A vaccination within the hepatitis B vaccination programme.
